# Factors associated with default from treatment among tuberculosis patients in nairobi province, Kenya: A case control study

**DOI:** 10.1186/1471-2458-11-696

**Published:** 2011-09-09

**Authors:** Bernard N Muture, Margaret N Keraka, Peter K Kimuu, Ephantus W Kabiru, Victor O Ombeka, Francis Oguya

**Affiliations:** 1 National Public Health Laboratory Services, Ministry of Health, P.O. Box 20750-00202, Nairobi, Kenya; 2 Department of Public Health, Kenyatta University, P.O Box 43844, Nairobi, Kenya; 3 National Leprosy and Tuberculosis Program (NLTP), Ministry of Health, P.O. Box 19265-00202, Nairobi, Kenya; 4 Department of Pathology, Kenyatta University, P.O Box 43844, Nairobi, Kenya

## Abstract

**Background:**

Successful treatment of tuberculosis (TB) involves taking anti-tuberculosis drugs for at least six months. Poor adherence to treatment means patients remain infectious for longer, are more likely to relapse or succumb to tuberculosis and could result in treatment failure as well as foster emergence of drug resistant tuberculosis. Kenya is among countries with high tuberculosis burden globally. The purpose of this study was to determine the duration tuberculosis patients stay in treatment before defaulting and factors associated with default in Nairobi.

**Methods:**

A Case-Control study; Cases were those who defaulted from treatment and Controls those who completed treatment course between January 2006 and March 2008. All (945) defaulters and 1033 randomly selected controls from among 5659 patients who completed treatment course in 30 high volume sites were enrolled. Secondary data was collected using a facility questionnaire. From among the enrolled, 120 cases and 154 controls were randomly selected and interviewed to obtain primary data not routinely collected. Data was analyzed using SPSS and Epi Info statistical software. Univariate and multivariate logistic regression analysis to determine association and Kaplan-Meier method to determine probability of staying in treatment over time were applied.

**Results:**

Of 945 defaulters, 22.7% (215) and 20.4% (193) abandoned treatment within first and second months (intensive phase) of treatment respectively. Among 120 defaulters interviewed, 16.7% (20) attributed their default to ignorance, 12.5% (15) to traveling away from treatment site, 11.7% (14) to feeling better and 10.8% (13) to side-effects. On multivariate analysis, inadequate knowledge on tuberculosis (OR 8.67; 95% CI 1.47-51.3), herbal medication use (OR 5.7; 95% CI 1.37-23.7), low income (OR 5.57, CI 1.07-30.0), alcohol abuse (OR 4.97; 95% CI 1.56-15.9), previous default (OR 2.33; 95% CI 1.16-4.68), co-infection with Human immune-deficient Virus (HIV) (OR 1.56; 95% CI 1.25-1.94) and male gender (OR 1.43; 95% CI 1.15-1.78) were independently associated with default.

**Conclusion:**

The rate of defaulting was highest during initial two months, the intensive phase of treatment. Multiple factors were attributed by defaulting patients as cause for abandoning treatment whereas several were independently associated with default. Enhanced patient pre-treatment counseling and education about TB is recommended.

## Background

Successful treatment of tuberculosis involves taking anti-tuberculosis drugs for at least six months. Kenya subscribes to the internationally accepted World Health Organization (WHO) strategy for TB control. In addition, the country has adopted the WHO recommended tuberculosis treatment regimes. Although treatment duration for new TB patients in Kenya was previously 8 months in total, a shorter 6-months regime was started in 2007 in Nairobi province and later expanded to cover the whole country by 2009. Consequently, duration of treatment within the study period was either six or eight months. In the first two months of treatment (intensive phase), a combination dose of rifampicin (R), isoniazid (H), pyrazinamide (Z) and ethambutol (E) (2RHZE) was used daily followed by either 6 months of ethambutol and isoniazid (6EH) for the 8 months regime; or 4 months of rifampicin and isoniazid (4RH) for the 6 months regime. During the intensive phase of treatment, patients collect drugs from facilities weekly while monthly collections are done during the continuation phase. The treatment regime for retreatment patients is 8 months and includes Streptomycin (S) in the first 2 months. Emphasis is made on Direct Observation of Treatment (DOT) by a health worker or other responsible persons, including household members or others with whom the patient has a close relationship, at least during the intensive phase of treatment.

Some patients fail to adhere to treatment and eventually default before completing the course. Patients whose treatment is interrupted for 2 consecutive months or more, as defined by WHO, are reported as 'Out of Control' at the end of treatment period. Poor adherence to treatment means that patients remain infectious for longer and are more likely to relapse or succumb to tuberculosis [1]. In addition, erratic or selective compliance to treatment and default could result in treatment failure, foster emergence of drug resistant tuberculosis [2,3] and may increase the cost of treatment. Due to serious consequences of default, some National TB Programs offer incentives and social support to ensure treatment compliance and completion to maximize the likelihood of cure, hence avoid adverse treatment outcomes and minimize the chances of developing drug resistance [4].

The WHO recommended Directly Observed Treatment Short Course (DOTS) strategy was introduced in Kenya in 1993 reaching 100% geographic coverage by 1997. In 2008, Kenya was ranked 13^th ^among the twenty two countries with high TB burden globally [5]. Incident cases of tuberculosis increased nine-fold from 11,625 cases in 1990 to 116,723 in 2007 [6] and is largely attributed to the HIV pandemic. Up to year 2006, treatment success rates stagnated around 80% in spite of the government's policy of free tuberculosis treatment in public health facilities (treatment success rate has since improved to 87% for the 2008 cohort as per the 2010 WHO Global TB Report). Default from treatment was among major hindrances to the achievement of the global target of successfully treating 85% of detected TB cases and remains a major challenge to its sustenance.

Nairobi has an estimated 3 million people, about 10% of the country's population but contributes about 20% of the national annual TB burden. Of the 116, 723 TB cases detected nationwide in 2006, 16.2% (18,906) were registered in Nairobi. It had the highest Case Notification Rate (CNR) at 652/100,000 population among the TB control regions. Further, it recorded the highest defaulter (Out of Control) rate of 16.7% for all cases *vs*. 9% nationally [6]. Defaulter retrieval mechanisms are generally weak in Kenya [7], including Nairobi. The high TB burden and defaulter rate were reasons why Nairobi was purposively selected for the study.

Adherence to long-term therapies is a multidimensional phenomenon determined by the interplay of five sets of factors (dimensions) namely; social and economic factors, health care team and system-related factors, condition-related factors, therapy-related and patient-related factors [8]. Improving treatment outcomes and designing effective interventions require understanding of the factors that prevent people from adhering and those that help in treatment completion. In Sub-Saharan Africa, several social and economic factors such as low income, lack of social support, low education, financial problems and inability to afford services [9,10] have been linked to TB treatment adherence. Older age, the male sex, inadequate knowledge, ignorance on need for treatment compliance and stigma [10-14] are among reported patient-related factors that influence default in the region. Reported health care system-related factors for default include poor service provider attitudes, negative attitude by tuberculosis patients towards the treatment centre, running out of drugs, access to health services and living near to treatment centre [10,13,14]. Side effects, drugs too strong, and feeling better [10,13,15] are among therapy related factors that influence TB treatment default while HIV co-morbidity is among the condition-related factor reported [11]. Studies on TB treatment default in Nairobi, and the country at large, have not been documented.

This study aimed to determine the duration TB patients stayed in treatment before default and the factors associated with default in Nairobi. Specifically, we evaluated the timing of treatment default among those who abandoned treatment; we examined the risk factors for treatment default; and, through interviews involving both structured and open-ended questions, we explored the health attitudes and beliefs associated with treatment default.

## Methods

A retrospective case-control study design was used utilizing both primary and secondary data.

### Study population

The study population comprised of the cohort of patients (adults and children) registered during the period January 2005 to March 2007 in 30 high-volume public TB treatment facilities (total number of treatment centers was about 200) distributed across all 8 health districts of Nairobi. About 14% of Nairobi's TB caseload was registered in these facilities. Cases were patients whose treatment was interrupted for 2 consecutive months or more (as defined by WHO), and reported 'Out of Control' (N = 945) while Controls were those who completed the treatment course (N = 1033).

### Sampling procedure

From the sampled facilities, all patients who defaulted (945) within the study period were enrolled. A total of 1033 controls were randomly selected from among 5659 patients who had completed treatment course. Cases and controls were matched for site (approximately equal number of each per treatment site). To enhance understanding of risk factors for default, a sample of 154 cases and 154 controls (total 308) aged 15 years and above (adults) were randomly selected from the study population from which 120 cases and all 154 controls were traced and interviewed. A minimum sample size of 113 cases and 113 controls was necessary to have 80% power of identifying an odds ratio of 3 or larger at the 95% level of statistical significance (two tailed)[16].

### Study procedure

Secondary data for the study population (cases and controls, N = 1978) were abstracted from TB treatment registers in selected treatment facilities using a facility questionnaire. These included demographic data (age, sex, residence, marital status) and medical and treatment data (treatment observer, patient and TB types, HIV status, treatment regimen, sputum smear microscopy results, the date treatment was started and ended and the treatment outcome).

To obtain primary data, sampled cases (n = 120) and controls (n = 154) were interviewed using a both open and close-ended structured questionnaire. Data on variables not routinely collected in treatment registers such as socioeconomic status, drug side effects, alcohol abuse and herbal medication use among others was collected. Also collected were data on factors pertaining to knowledge about TB including prior attendance to a TB health education session, suspecting TB at onset, knowledge about TB transmission and duration of treatment, awareness that TB is curable and history of TB among household members or friends.

### Statistical analysis

Analysis was carried out for both secondary and primary data, stratified for cases and controls. Epi Info for Windows, Version 3.3.2 and SPSS 11.5 statistical software packages were used for data analysis. Analysis of contingency tables to determine associations was used. Two-tailed Yates-corrected chi-square or Fisher exact test (for cells with values less than 5) was used to assess categorical variables. Odds ratios were used as measure of association and corresponding 95% confidence intervals calculated using Taylor (T) series. Variables significant at the two-tailed 0.1 level during univariate analysis were included in multivariate logistic regression model. Kaplan-Meier survival analysis method was used to determine probabilities of defaulters continuing with the treatment program over different durations.

### Ethical Consideration

The study was retrospective and did not involve any experimental procedures on patients. However, research and ethical clearance to conduct the study was sought and obtained from Kenyatta University and a research permit was granted by Kenya's Ministry of Science and Technology (REF: MOST 13/001/37C795/2 of 29^th^, November 2007). TB is strongly associated with HIV/AIDS leading to stigmatization and is difficult to discuss in public. Informed consent was obtained from patients and confidentiality and anonymity was assured. Names and addresses of patients were collected only for the purposes of follow-up.

## Results

### Characteristics of study population

A total of 1978 cases and controls (945 cases and 1033 controls) were enrolled. The mean age was 31.2 years (range 1-80) for Cases and 29.5 years (range 1-78) for Controls. Among cases, 59.4% (561) were male and 40.6% (384) female. Of the 1033 controls 53% (547) were male and 47% (486) female. Treatment observation for 679 (71.9%) of cases and 785 (76%) of controls was by household members. HIV testing for TB patients through the Diagnostic Testing and Counseling (DTC) approach had been conducted for 1569 (79.3% testing rate) of which 863 (55.0%) were co-infected. Co-infection among cases was 61.5% (405) compared to 50.3% (458) for controls. Majority of patients, 78.9% (746) cases and 79.4% (820) controls had pulmonary TB (Table 1).

**Table 1:** Characteristics of study population (Source: secondary data, n = 1978)

	Cases (n = 945)	Controls (n = 1033)			
Characteristic	N (%)	N (%)	Total (%)	OR (95% CI)	P value
**Age group (Years)**					
Children (< 14)	68 (7.2)	102 (9.9)	170 (8.6)	1	0.6
Adolescents (14-19)	36 (3.8)	61 (5.9)	97 (4.9)	0.89 (0.53-1.48)	0.03
Young adults (20-40)	675 (71.4)	702 (68.0)	1377 (69.6)	1.44 (1.04-1.98)	0.12
Middle-aged adults (41-59)	139 (14.7)	153 (14.8)	292 (14.8)	1.36 (0.93-2.0)	0.004
Elderly(≥ 60)	27 (2.9)	15 (1.5)	42 (2.1)	2.70 (1.42-5.91)	
**Gender**					
Male	561 (59.4)	547 (53.0)	1108 (56.0)	1.3	0.01
Female	384 (40.6)	486 (47.0)	870 (44.0)	(1.08-1.55)	
**Treatment observer**					
Health care worker	266 (28.1)	248 (24.0)	514 (26.0)	1.24	0.05
Household member	679 (71.9)	785 (76.0)	1464 (74.0)	(0.999-1.52)	
**Co-infected with HIV ^β^**					
Yes	405 (61.5)	458 (50.3)	863 (54.9)	1.57	< 0.001
No	254 (38.5)	452 (49.7)	708 (45.1)	(1.31-1.97)	
**Initial AFB sputum result ^Ψ^**					
Smear-positive	391 (55.2)	454 (55.0)	845 (55.1)	1.01	0.96
Smear-negative	317 (44.8)	372 (45.0)	689 (44.9)	(0.83-1.24)	
**Type of Tuberculosis**					
Extra Pulmonary	199 (21.1)	213 (20.6)	412 (20.8)	1.03	0.89
Pulmonary	746 (78.9)	820 (79.4)	1566 (79.2)	(0.82-1.28)	
**Patient type**					
Return After Default (RAD)	27 (2.9)	15 (1.5)	42 (2.1)	2	0.02
New/Relapse	918 (97.1)	1018 (98.5)	1936 (97.9)	(1.14-4.09)	

**^β ^**for patients whose HIV status was available

**^Ψ ^**for patients whose sputum smear was done

OR - Odds Ratio, CI - Confidence Intervals

### Duration of treatment before default

**Figure 1:**
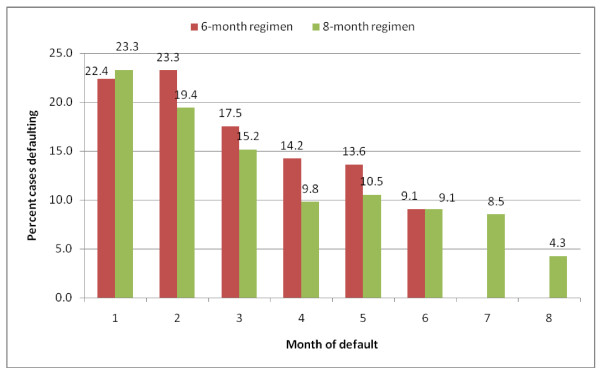
**Proportion of defaulters (cases) by time of default (months) for both six (N = 359) and eight-month (N = 586) regimens in Nairobi**.

The rate of defaulters abandoning treatment for both 6 and 8-month regimes was high during initial months of treatment and generally decreased each consecutive month (Figure 1). Of 945 patients for whom the treatment course was not completed (cases), 22.7% (215) and 20.4% (193) abandoned treatment within the first and second months of treatment respectively, thus 43.1% (408) during the intensive phase. Of 359 cases on the 6-month regime, 45.7% (164) defaulted within the first two months of treatment comparable to 42.7% (250 out of 586) of cases on the 8-month regime.

**Figure 2:**
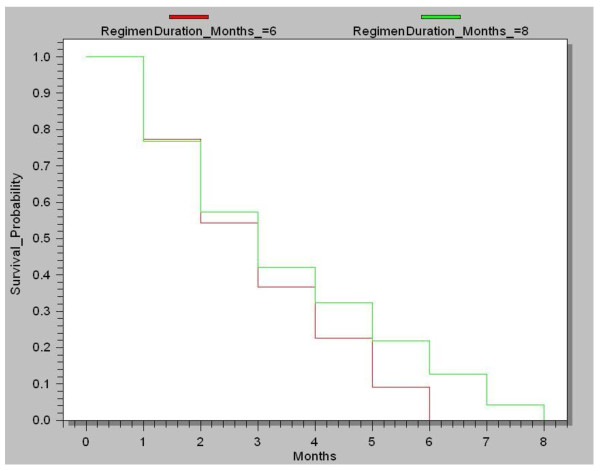
**Kaplan-Meier curve for TB treatment defaulters (cases) remaining in treatment over time (for both six (N = 359) and eight-month (N = 586) regimens in Nairobi**.

Figure 2 shows the Kaplan-Meier survival analysis for the probability of defaulters (cases) on the 6 and 8-months regimes staying in treatment over different lengths of time. Cases on the 6-month regime had 77.4% and 54.2% chance of staying in treatment in the first and second months respectively (this was 76.7% and 57.3% respectively for cases on the eight-month regime). Among 391 cases who were sputum smear positive for Mycobacterium tuberculosis at the commencement of treatment, 187 (47.7%) defaulted before bacteriological conversion was confirmed.

### Characteristics of sample population

To enhance understanding of risk factors for default, a sample of 308 adults (154 cases and 154 controls) were randomly selected from the study population. All sampled controls were traced and participated in the interviews. Of the 154 cases sampled, 120 (78.0%) were interviewed while information obtained during tracing for 23(14.9%) was that they had died while 11(7.1%) could not be traced. Data obtained from the interviews (N = 274) was used to identify factors patients attributed to their default and in univariate analysis to determine factors associated with default.

Of the 274 patients interviewed, 153 (55.8%) had education lower than secondary level among which the majority 86 (71.4%) were defaulters and 67 (43.2%) were controls. A total of 181 (66.1%) patients interviewed were unemployed. Among the defaulters, 82 (68.2%) were unemployed comparable to 99 (64.3%) for controls. Monthly income for 110 (92.1%) cases and 109 (71.3%) controls was below 10,000 Kenya shillings (approximately 140 US dollars) per month, (the lower income-level for the Kenyan urban population). Mean household population was 4.7 persons per household for cases and 5.4 for controls and majority (77.8%) lived in single-roomed houses. Duration of residency in treatment locality varied widely but 72.3% of patients had been residents in the treatment locality for more than two years (Table 2).

**Table 2:** Univariate analysis of association of social demographic and socioeconomic factors with treatment default outcome (n = 274)

	Cases (n = 120)	Controls (n = 154)		
Factors	N (%)	N (%)	OR (95% CI)	P value
**Marital status**				
Single/Widowed/Separated	69 (57.3)	69 (45.0)	1.67	0.05
Married	51 (42.7)	85 (55.0)	(1.01-2.66)	
**Employment status**				
Unemployed	82 (68.2)	99 (64.3)	1.2	0.59
Employed	38 (31.8)	55 (35.7)	(0.71-2.04)	
**Level of education**				
Non-formal/Primary	86 (71.4)	67 (43.2)	3.28	< 0.001
Secondary/Post secondary	34 (28.6)	87 (56.8)	(1.96-5.55)	
**Duration of residence in treatment area**				
≤ 2 years	47 (39.2)	29 (19.3)	2.78	< 0.001
> 2 years	73 (60.8)	125 (81.2)	(1.57-4.98)	
**Alcohol abuse**				
Yes	44 (36.9)	13 (8.6)	6.28	< 0.001
No	76 (63.1)	141 (91.4)	(3.15-12.45)	
**Monthly Income**				
< KShs 10,000	110 (92.1)	109 (71.3)	4.5	< 0.001
≥ KShs 10,000	10 (7.9)	45 (28.7)	(1.9-11.4)	
**Household Size**				
Persons, mean (range)	4.7 (1-18)	5.4 (1-18)	t = 1.8	0.07
**House size**				
≤ double rooms	112 (93.3)	134 (87.0)	2.09	0.15
> double rooms	8 (6.7)	20 (13.0)	(0.87-4.83)	
**Knowledge on TB ^α^**				
Inadequate	49 (40.5)	32 (21.1)	2.6	< 0.001
Adequate	91 (59.6)	122 (78.9)	(1.48-4.4)	
**Experienced stigmatization**				
Yes	67 (55.8)	55 (35.7)	2.28	0.002
No	53 (44.2)	99 (64.3)	(1.35-3.7)	
**Experience drug side-effects**				
Yes	72 (59.6)	83 (53.6)	1.28	0.39
No	48 (40.4)	71 (46.4)	(0.78-2.09)	
**Herbal Medication used during therapy**				
Yes	32 (26.7)	5 (3.3)	10.76	< 0.001
No	88 (73.3)	149 (96.7)	(4.0-28.63)	
**Waiting time for services at facility**				
≥ 1 hour	41 (34.2)	28 (18.2)	2.34	0.005
< 1 hour	79 (65.8)	126 (81.8)	(1.32-4.16)	

**^α^**Questions on transmission, treatment, cure and past experience of TB at household used to assess knowledge on TB

OR - Odds Ratio, CI - Confidence Intervals

### Risk factors for default

**Figure 3:**
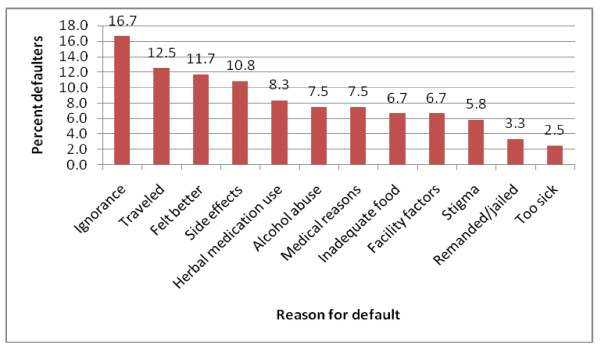
**Reasons defaulting patients attributed to their default from TB treatment in Nairobi (N = 120)**.

Through an open ended question, patients who defaulted from treatment were asked to give one most important reason for their default. The frequencies of cited reasons are shown in Figure 3 whereby ignorance was the most cited. Further, univariate analysis was performed to identify factors associated with default for the study population (Table 1) and sample population (Table 2) and multiple factors had significant association. Results of assessment on factors pertaining to TB knowledge are shown in Table 3. In multivariate logistic regression of the primary data, HIV co-infection, history of previous default, male sex, herbal medication use, inadequate knowledge about TB, alcohol abuse and low socioeconomic status were independently associated with default (Table 4). Results show default from TB treatment in the region is influenced by the interplay of all 5 dimensions of adherence to long-term therapy.

**Table 3:** Assessment of factors pertaining to TB knowledge

	Cases (n = 120)	Controls (n = 154)		
Factors	N (%)	N (%)	OR (95% CI)	P value
**History of TB in household**				
No	90 (75.3)	115 (74.7)	1.02	0.96
Yes	30 (24.7)	39 (25.3)	(0.53-1.79)	
				
**TB suspected at onset**				
No	68 (56.6)	81 (52.6)	1.18	0.6
Yes	52 (43.4)	73 (47.4)	(0.72-1.92)	
				
**Had Prior TB health education**				
No	55 (45.9)	29 (19.1)	3.65	< 0.001
Yes	65 (54.1)	125 (80.9)	(2.08-6.25)	
				
**Is TB curable?**				
No/don't know	37 (31.0)	5 (3.2)	13.28	< 0.001
Yes	83 (69.0)	149 (96.8)	(5.04-35.5)	
				
**Knowledge on Duration of TB cure**				
Don't know	24 (20.0)	5 (3.2)	7.45	< 0.001
6-8 months	96 (80.0)	149 (96.8)	(2.72-20.4)	
				
**Knowledge on TB transmission**				
Don't know	12 (9.8)	11 (7.2)	1.44(0.58-3.35)	0.6
From infected via cough, etc	108 (90.2)	143 (92.8)		
				
**TB Perceived like any other disease**				
No	96 (80)	89 (57.8)	2.92	< 0.001
Yes	24 (19)	65 (42.2)	(1.66-5.39)	

**Table 4:** Multivariate logistic regression analysis of factors independently associated with default

Factor	Adjusted odds ratio	95% Confidence intervals	P-value
HIV co-infection	1.56	1.25-1.94	0.001
History of previous default	2.33	1.16-4.68	0.02
Gender (Male/Female)	1.43	1.15-1.78	0.001
Age	1.01	1.00-1.02	0.18
DOTS observer (Health Care Worker/Household member)	1.20	0.94-1.53	0.14
Herbal medication use	5.70	1.37-23.7	0.02
Education level lower than Secondary	2.29	0.87-6.0	0.09
Inadequate knowledge on TB	8.67	1.47-51.3	0.02
Alcohol abuse	4.97	1.56-15.9	0.01
Monthly income lower than KShs. 10,000	5.57	1.07-30.0	0.04
Stigmatization	1.92	0.69-5.39	0.21

#### Health care and system-related factors

Of 120 patients who did not complete the treatment course, 15(12.5%) attributed their default to traveling away from treatment localities, consequently missing scheduled appointment or running short of drugs. Unfavorable health facility factors, including unavailability of drugs on all scheduled clinic days, failure by health providers to: offer health education on TB, articulate the need for treatment compliance, and appropriately manage drug side-effects were also cited as reasons for default. Other factors included limited access to health care and waiting too long for services. Waiting for services for more than 1 hour was significantly associated with default (OR 2.34, CI 1.32-4.16) on univariate analysis but the effect was absent after controlling for confounding (Table 2). Some unfavourable health-care personnel attitudes cited included being unfriendly, unsympathetic and lack of dignity.

#### Social and economic factors

Among defaulters, 8 (6.6%) cited inadequate food as reason for their default. From primary data, being unmarried (OR 1.67; 95% CI 1.01-2.66), education lower than secondary level (OR 3.28; 95% CI 1.96-5.55), less than two years residency in treatment locality (OR 2.78; 95% CI 1.57-4.98), and stigma (OR 2.28; 95% CI 1.35-3.7) were associated with default. Use of herbal medication (OR 5.7; 95% CI 1.37-23.7) and low income (OR 5.57; 95% CI 1.07-30.0) were predictive for default.

#### Patient-related factors

Ignorance on need for treatment compliance, coupled with inadequate knowledge about tuberculosis was cited by 20 out of 120 (16.7%) cases and was the most frequent reason attributed to default. Assessment of some factors pertaining to knowledge about TB (Table 3) showed that among cases; 90 (75.3%) had no history of TB in their households compared with 115 (74.7%) controls, 68 (56.6%) cases *vs*. 81 (52.6%) controls did not suspect TB at onset, while 55 (45.9%) cases compared to 29 (19.1%) controls had never read any material or attended any TB health education session before their illness. Among cases, 35 (31.0%) were unaware TB is curable compared to 5(3.2%) controls, while 24 (20%) cases compared to 5 (3.2%) controls did not know the duration for TB treatment. Prior attendance to a TB health education session, awareness that TB is curable and knowledge on duration of treatment, as well as perceiving TB as any other disease contributed to better adherence.

Univariate analysis of secondary data indicated young adults (OR 1.43; 95% CI 1.04-1.98) and the elderly (OR 2.89; 95% CI 1.42-5.91) were at a higher risk of defaulting compared to children. Further, inadequate knowledge on TB (OR 8.67; 95% CI 1.47-51.3) and the male gender (OR 1.43; 95% CI 1.15-1.78) were independently associated with default on multivariate analysis.

#### Condition-related factors

Recurring use of alcohol (alcohol abuse) and consequently forgetting to take drugs and eventually defaulting was cited by 9 (7.5%) of cases. Medical reasons given for default included psychiatric conditions (3), development of multi-drug resistance (2), TB misdiagnosis (1), heart problems (1) and pregnancy (1). From primary data, alcohol abuse, defined as recurring use of alcoholic drinks despite the negative consequences, was found a predictive factor for default (OR 4.97; CI 1.56-15.9). Further, secondary data indicated that co-infection with HIV/AIDS (OR 1.57; 95% CI 1.28-1.93) and history of default (OR 2.16; 95% CI 1.14-4.09) were also predictive for default (Table 1 and 4).

#### Therapy-related factors

Anti-tuberculosis drug side-effects were attributed by 13 (10.8%) defaulters as cause for their default. However, on univariate analysis, side effects were not significantly associated with default. Feeling better after medication for a while (and perceiving it as cure) was cited by 14 (11.7%) defaulters as reason why they stopped taking of drugs.

## Discussion

A substantially high number of patients abandoned treatment soon after initiation of treatment. Consequently, many of the patients who were sputum smear positive for *Mycobacterium tuberculosis *(infectious) did not stay long enough in treatment to convert to smear negative. The study findings indicated that multiple factors influenced default in Nairobi including; inadequate knowledge about TB, HIV co-infection, opting for herbal medication, previous default and low socioeconomic status. Early default during treatment is likely to lead to adverse outcomes (treatment failure, death and drug resistance). Although similar findings have been reported among Brazilian children and in Hong Kong [17,18], patients in several countries in the Sub-Saharan Africa and in Singapore have been reported to default more frequently during the continuation phase [10,11,19].

Majority of patients (56.6% of cases and 52.6% of controls) did not suspect TB at onset and were probably unaware of the disease before they presented themselves to the health facilities. On diagnosis such patients should receive sufficient explanation of their disease, made to understand the treatment requirements, likely side effects to be encountered when using anti-TB drugs and the need to comply with treatment. Tuberculosis caseload has steadily risen over the years in Kenya while HIV/AIDS pandemic is a declared national disaster. Despite the rise in TB disease burden, recruitment of health workers in the public health sector has been restricted in the last 15 years until recently. Health workers who left the service within that period due to natural attrition or relocated to work in other countries were therefore not replaced resulting to reduced workforce. Due to subsequent high workloads for health personnel at health facilities in the country, pre-treatment health education is unlikely to be sufficient compounding to the poor defaulter tracing mechanisms. Indeed, a substantially high number of defaulters attributed their default to ignorance and inadequate knowledge about TB. Inadequate knowledge was found a significant factor for default similar to findings in Madagascar [14].

Another possible explanation for the early default could be lack of adequate food, as cited by some defaulters. Patients on tuberculosis treatment usually experience an increased appetite. Although a good sign indicating clinical response, to the low income group where access to food is a problem, inadequate food may pose a challenge to treatment adherence. Further, the weekly collection of drugs comes with transport costs to and from treatment centers. Majority (66.1%) of our study patients were unemployed indicating resources for transport and other opportunity costs could have been a challenge.

Drugs used during the intensive phase rapidly reduce the number of tubercle bacilli (bacillary load) in the body and patients usually feel better shortly after initiation of treatment. Inadequately counseled patients may mistake the feeling of improvement to cure, thus stop medication early. Feeling better was cited among reasons for default and has similarly been reported in other studies as cause for default [10,13,15]. Adequate patient education and counseling at initiation of treatment is therefore important and could mitigate early default.

In developing countries, low socioeconomic status may put patients in the position of having to choose between competing priorities. Such priorities frequently include demands to direct the limited resources available to meet the basic needs. In Kenya, the government supports treatment of tuberculosis by availing free diagnostic services and drugs, but other hidden costs such as transport and opportunities lost during treatment exist. The health budget is usually overstretched and resources for social support are scarce or unavailable. Similar to findings in some Sub-Saharan African countries [9,10], socioeconomic factors such as low income and low education were linked to TB treatment default.

Undocumented findings indicate a big number of informal health practitioners (herbalists, traditional healers and medicine men) are practicing in Nairobi in competition with formal practitioners. As a result, some patients opt to use the herbal medication in place of the recommended TB drugs. We report use of herbal medication a risk factor for default, which has not previously been reported. Traditional healers function as social workers and psychologists in their community and are easily accessible. The efficacy of their herbal remedies may be questionable but their knowledge of the local dynamics is real. They are highly revered and respected in the society, especially where illness is perceived to result from witchcraft (as TB and HIV are sometimes perceived in Africa). Within the communities of the study population, 50% of patients indicated tuberculosis is perceived as HIV/AIDS while others perceive it as inherited, non-curable or resulting from a curse, taboo or witchcraft. Patients' knowledge and beliefs about their illness, motivation to manage it and consequences of poor adherence interact to influence adherence behaviour. There is clear evidence of the effect on adherence by culturally influenced attitudes and beliefs about tuberculosis and its treatment. Cultural factors are associated with misinformation about the medical aspects of the disease and stigmatization of persons with tuberculosis. In Southern Africa, Public health specialists have enlisted sangoma (traditional healers) in the fight against the spread of HIV/AIDS. Unlike HIV, TB can be cured by use of and adherence to the WHO recommended regimes, a fact that the herbalists should be sensitized on and engaged in TB patient referrals. Besides easy access to herbal medicines, the side effects associated with TB drugs if inadequately managed could be reasons why patients opt for herbal medication. A number of patients attributed their default to the side-effects of anti-tuberculosis drugs. Care givers should receive continuous medical education to be conversant in the management of these side effects to minimize the chances of patients opting to herbal medication.

HIV co-infected patients have been reported to have twice the risk of defaulting during the intensive phase of TB treatment compared to HIV negative patients in Nigeria [11]. Similarly, poorer TB treatment success rate for HIV positive patients among re-treatment patients has previously been reported in Nairobi [20]. In our study, HIV co-morbidity was found a predictive factor for default. Many TB patients in the study (54.9%) were also co-infected with HIV. The co-infected patients often attend separate clinics or facilities for TB and HIV care services, thus increasing transport and other opportunity costs. The side-effects profile of TB chemotherapy is magnified in patients with concurrent HIV treatment [21]. Besides, combining anti-retroviral and TB drugs means taking many tablets daily and can be difficult and challenging to a patient. That patients with HIV co-morbidity are significantly more likely to default is sufficient evidence that HIV and TB care should be integrated. As a step towards integration of TB and HIV care, the National tuberculosis control program (NTP) and the National AIDS and Sexually Transmitted Diseases (STD) Control Program (NASCOP) in Kenya have a policy for screening TB patients at treatment sites for HIV and vice versa. This needs to be scaled up.

Recurring use of alcohol (alcohol abuse) leads to forgetting the taking of drugs and eventual default. In Nairobi's informal settlements where majority of the study population lived, cheap local brews are very common and sold in poorly ventilated and congested premises. Indulgence in these easily affordable alcoholic brews was witnessed among some TB patients during tracing. Such patients pose a serious threat of transmitting TB to other patrons and are at an increased risk of defaulting. Besides, alcohol is injurious to the liver, potentiating the hepatic effects of anti-tuberculosis drugs. Alcohol combined with anti-TB drugs may lead to a greater risk of liver damage.

Whereas a good patient-provider relationship may improve adherence, many health care system-related factors that have a negative effect in Sub-Saharan Africa exist. These include poor service provider attitudes, negative attitude by tuberculosis patients towards the treatment centre, running out of drugs and poor access to health services [10,13,14]. Without proper prior arrangements, patients who travel away from treatment centers are likely to run out of drugs. About 12% of the defaulters attributed their default to having travelled away from treatment locality during which they ran out of drugs. Unfavorable health facility factors cited included unavailability of drugs on scheduled appointment dates, failure by health provider to offer adequate health education about TB treatment (probably due to overburdened health care providers and weak capacity of the system to educate patients and provide follow-up) and waiting too long for services. Unfavorable health care personnel attitudes including being unfriendly, unsympathetic and lack of dignity were also cited.

The study excluded patients registered in private TB treatment facilities as well as patients in low volume public facilities. This has the potential of affecting inference of the study findings to the whole of Nairobi region. Further, some sampled defaulters were not traced while others had died but not notified to their treatment facilities and were thus miscategorized as 'Out of Control' rather than 'died' in the treatment registers and were thus sampled as defaulters. These may have lead to some bias in the findings and is a potential limitation of the study. To address this bias, we interviewed a slightly higher number of cases and controls than the calculated minimum sample size required.

## Conclusions

The rate of default from TB treatment occurs most frequently during early months of treatment in Nairobi with a good number of smear positive patients defaulting without confirmation of bacteriological conversion. The early default could be a result of inadequate pre-treatment health education and counseling and poor defaulter tracing mechanism resulting from overworked health care personnel, feeling better after medication for a while and socioeconomic factors including inadequate food and opportunity costs. Multiple factors influence default. The most frequent reasons for default cited by patients who did not complete the treatment course included ignorance about need for treatment compliance coupled with inadequate knowledge about TB and traveling outside treatment areas, consequently missing clinic appointment and running out of drugs. Predictive factors for default were inadequate knowledge about TB, herbal medication use, low income, alcohol abuse, previous default, HIV co-infection and the male sex.

Enhanced patient pre-treatment counseling and education on TB is recommended. This should include:

1. Emphasis on the importance of completing treatment without interruption regardless of feeling better, travelling away and side effects. Patients need to be educated on possible anti-TB drugs side-effects and supported to handle them. Patients travelling away from treatment centres should be informed of options available to ensure that they will not run short of drugs. In addition, DOT supporters could be allowed to collect the drugs for very sick patients. Targeted intervention aimed at assuring adherence in persons abusing alcohol is also recommended.

2. Assuring patients that TB is curable upon using correct TB drugs as prescribed by health workers. More studies are needed to identify why patients result to non-conventional medicines, including herbal medication in Nairobi. However, the role of informal health providers in TB control needs to be recognized. Health care providers need to manage side effects which may be among reasons patients opt to herbal medication.

In addition to enhanced patient counseling and education, patient-friendly TB care services as well as improved care for TB/HIV co-infected patients (including integration of services), is required.

## Competing interests

The authors declare that they have no competing interests.

## Authors' contributions

BNM initiated the study concept and design, supervised data collection, participated in patient tracing and interviews, cleaned, coded, entered and analyzed data and drafted the manuscript. MNK was the main supervisor of the study and was involved from the concept, design, data acquisition and interpretation of the study findings. PKK facilitated in data acquisition, critically reviewed the study design to conform to National TB program guidelines and terminology, provided critical interpretation of results and drafted the manuscript. EWK participated in critical revision of study design and supervision of the study. VO was involved in data acquisition and drafting of the manuscript. FO participated in the design of the study, guidance on statistical analysis and results presentation. All authors read and approved the final manuscript.

## Pre-publication history

The pre-publication history for this paper can be accessed here:

http://www.biomedcentral.com/1471-2458/11/696/prepub

## References

[B1] Salla Munro A Simon Lewin A Helen Smith J Mark Engel E Atle Fretheim Jimmy Volmink Patient Adherence to Tuberculosis Treatment: A Systematic Review of Qualitative Research PLoS Med 2007 4 7 e238 ^© ^2007 10.1371/journal.pmed.0040238 PMC1925126 17676945

[B2] Pablos-Mendez A Knirsch CA Barr RG Lerner BH Frienden TRL Non-adherence in tuberculosis treatment: Predictors and consequences in New York City American Journal of Medicine 1997 102 164 170 10.1016/S0002-9343(96)00402-0 9217566

[B3] Hong Kong Chest Service/British Medical Research Council Controlled trial of 2, 4, and 6 months of pyrazinamide in 6-month three times weekly regimens for smear-positive pulmonary tuberculosis, including an assessment of a combined preparation of isoniazid, rifampin and pyrazinamide: results at 30 months Am Rev Respir Dis 1991 143 700 706 1901199 10.1164/ajrccm/143.4_Pt_1.700

[B4] Jakubowiak WM Bogorodskaya EW Borisov ES Danilova DI Kourbatova EK Risk factors associated with default among new pulmonary TB patients and social support in six Russian regions The International Journal of TB and Lung Disease 2007 11 1 46 53 17217129

[B5] WHO Global tuberculosis control, surveillance, planning, financing report 2008

[B6] MOH NLTP Annual report 2007

[B7] MOH National Leprosy and Tuberculosis Programme, Strategic Plan, 2006-2010

[B8] WHO Adherence to long-term therapies, Evidence for Action 2003 Chapter 5 1 11 15

[B9] Dodor EA Afenyandu GY Factors associated with tuberculosis treatment default and completion at Effia-Nkwanta Regional Hospital in Ghana Trans R Soc Trop Med Hygiene 2005 99 11 827 832 10.1016/j.trstmh.2005.06.011 16102791

[B10] Demissie M Kabede D Defaulting from tuberculosis treatment at the Addis Ababa TB Centre and factors associated with it Ethiopian Medical Journal 1994 32 2 97 106 8033883

[B11] Daniel OJ Oladapo OT Alausa OK Default from treatment programme in Sagamu, Nigeria Nigeria Journal of Medicine 2006 15 1 63 7 10.4314/njm.v15i1.37119 16649455

[B12] Kaona FAD Tuba M Siziya S Sikaona S An assessment of factors contributing to treatment adherence and Knowledge of TB transmission among patients on TB treatment BMC Public Health 2004 4 68 10.1186/1471-2458-4-68 15625004 PMC545081

[B13] Wasonga J Factors contributing to tuberculosis treatment defaulting among slum dwellers in Nairobi, Kenya, International congress on drug therapy in HIV 2006 The Gardiner-Caldwell Group Ltd 310

[B14] Comolet TM Rakotomalala R Rajaonarioa H Factors determining compliance with tuberculosis treatment in urban environment, Tamatave, Madagascar International Journal of Tuberculosis and Lung diseases 1998 2 11 891 897 9848609

[B15] Jaiswal A Singh V Ogden JA Porter JDH Sharma PP Sarin R Arora VK Jain RC Adherence to tuberculosis treatment: Lessons from the urban setting of Delhi, India Journal of Tropical Medicine and International Health 2003 8 7 625 10.1046/j.1365-3156.2003.01061.x 12828545

[B16] Lemeshow S Hosmer DW Klar J Lwanga SK Adequacy of sample size in health studies John Wiley & Sons, WHO/HST/ESM/86, 1986, rev 1

[B17] Oliveira VL da Cunha AJ Alves R Tuberculosis treatment default among Brazilian children International Journal on Tuberculosis and Lung Diseases 2006 10 8 864 9 16898370

[B18] Chan-Yeung M Noertjojo K Leung CC Chan SL Tam CM Prevalence and predictors of default from tuberculosis treatment in Hong Kong Hong Kong Medical Journal 2003 9 4 263 8 12904614

[B19] Chee CBE Boudville IC Chan SP Zee YK Wang YT Patient and disease characteristics and outcome of treatment defaulters from the Singapore TB control unit-a one-year retrospective survey International Journal of Tuberculosis and Lung diseases 2000 4 6 496 503 10864179

[B20] Chakaya JM Kibuga D Nganga L Githui WA Mansoer JR Gakiria G Kwamanga D Maende J Tuberculosis re-treatment outcomes within public service in Nairobi, Kenya East African Medical Journal 2002 79 10.4314/eamj.v79i1.8918 12380864

[B21] Fry RS Khoshnood K Vdovichenko E Granskaya J Sazhin V Shpakovskaya L Barriers to completion of tuberculosis treatment among prisoners in St. Petersburg, Rusia International Journal on Tuberculosis and Lung Diseases 2005 9 1027 33 16158896

